# Progress in Investigational Agents Targeting Serotonin-6 Receptors for the Treatment of Brain Disorders

**DOI:** 10.3390/biom13020309

**Published:** 2023-02-07

**Authors:** Ramakrishna Nirogi, Pradeep Jayarajan, Anil Shinde, Abdul Rasheed Mohammed, Venkata Ramalingayya Grandhi, Vijay Benade, Vinod Kumar Goyal, Renny Abraham, Venkat Jasti, Jeffrey Cummings

**Affiliations:** 1 Suven Life Sciences Limited, Serene Chambers, Road-5, Avenue-7, Banjara Hills, Hyderabad 500034, Telangana, India; 2 Chambers-Grundy Center for Transformative Neuroscience, Department of Brain Health, School of Integrated Health Sciences, University of Nevada, Las Vegas, NV 89154, USA

**Keywords:** clinical trials, 5-HT_6_ receptor antagonist, avisetron, cerlapirdine, idalopirdine, intepirdine, landipirdine, latrepirdine, SAM-760, masupirdine, schizophrenia, Alzheimer’s disease, dementia, neuropsychiatric symptoms, psychosis, agitation

## Abstract

Serotonin (5-HT) plays an important role in the regulation of several basic functions of the central and peripheral nervous system. Among the 5-HT receptors, serotonin-6 (5-HT_6_) receptor has been an area of substantial research. 5-HT_6_ receptor is a G-protein-coupled receptor mediating its effects through diverse signaling pathways. Exceptional features of the receptors fueling drug discovery efforts include unique localization and specific distribution in the brain regions having a role in learning, memory, mood, and behavior, and the affinity of several clinically used psychotropic agents. Although non-clinical data suggest that both agonist and antagonist may have similar behavioral effects, most of the agents that entered clinical evaluation were antagonists. Schizophrenia was the initial target; more recently, cognitive deficits associated with Alzheimer’s disease (AD) or other neurological disorders has been the target for clinically evaluated 5-HT_6_ receptor antagonists. Several 5-HT_6_ receptor antagonists (idalopirdine, intepirdine and latrepirdine) showed efficacy in alleviating cognitive deficits associated with AD in the proof-of-concept clinical studies; however, the outcomes of the subsequent phase 3 studies were largely disappointing. The observations from both non-clinical and clinical studies suggest that 5-HT_6_ receptor antagonists may have a role in the management of neuropsychiatric symptoms in dementia. Masupirdine, a selective 5-HT_6_ receptor antagonist, reduced agitation/aggression-like behaviors in animal models, and a post hoc analysis of a phase 2 trial suggested potential beneficial effects on agitation/aggression and psychosis in AD. This agent will be assessed in additional trials, and the outcome of the trials will inform the use of 5-HT_6_ receptor antagonists in the treatment of agitation in dementia of the Alzheimer’s type.

## 1. Introduction

Serotonin-5-hydroxytryptamine (5-HT) or enteramine was discovered in the 1930s [1], and its presence in the brain was identified in the year 1953 [2]. Since then, advancements have been made in characterizing 5-HT and its receptors. 5-HT functions as an inhibitory monoamine neurotransmitter, a hormone, and a mitogen that plays an important role in the regulation of several basic functions of the central and peripheral nervous system [3]. The 5-HT transporter regulates the availability of 5-HT in the synapse. 5-HT mediates its functions through seven distinct families of receptors (5-HT_1-7_), which comprise 14 distinct receptor subtypes. Most of the 5-HT receptor types are G-protein coupled receptors (GPCRs), except for the 5-HT_3_ receptor, which is a ligand-gated cation channel. GPCRs can modulate diverse downstream signaling pathways, and this may partly explain the critical role of serotonin in controlling/modulating multiple physiological functions [4,5].

Only a fraction (~5%) of the total amount of 5-HT is produced in the brain [3,6], but 5-HT and 5-HT receptors play an important role in the regulation of many brain functions. In addition, the 5-HT system also interacts with other neurotransmitter systems in the brain [7]. Dysregulation of the 5-HT and/or 5-HT receptors has been implicated in the pathogenesis of several disorders including, but not limited to depression, anxiety, Alzheimer’s disease (AD), schizophrenia, Parkinson’s disease, and sleep disorders [8,9,10]. The majority of drugs (~40%) approved for use in humans for the treatment of various disorders target the 5-HT system [11]. The distribution of 5-HT receptors in the brain and diverse signaling pathways involved make the 5-HT system an important potential target for therapeutic interventions. The development and characterization of gene knock-out animals; expanding knowledge of receptor expression levels, specific localization, distribution and function; and progress in receptor subtype-specific agents comprise significant advancements in the area of 5-HT research. Considering the widespread distribution and functions of the serotonergic system, targeting the 5-HT receptors with specific characteristics and localization may define a drug development pathway. One of the 5-HT receptors—serotonin-6 (5-HT_6_) receptor—has been an area of substantial research. 

The 5-HT_6_ receptor was first cloned and characterized from rat striatum by Monsma and colleagues in 1993 [12]. The human 5-HT_6_ receptor was cloned and characterized in 1996 by Kohen and colleagues [13]. Detailed review of the discovery and characterization of the 5-HT_6_ receptor has been previously reported [14,15,16]. Briefly, the 5-HT_6_ receptor is a GPCR and is coupled to Gs/adenylyl cyclase pathway (adenylyl cyclase3 isoform) [16,17]. 5-HT_6_ receptors are uniquely localized in the brain regions, including the olfactory tubercle, cerebral cortex, nucleus accumbens, striatum, hippocampus, thalamus, and cerebellum [12,18], providing an opportunity to identify new treatments modulating functions of specific brain regions. Although peripheral 5-HT_6_ receptors expression has been reported in rodents, their expression is faint [16]. Thus, selective targeting of 5-HT_6_ receptors may avoid unwanted side effects arising from other body systems (peripheral as well as other brain regions). In this report, we review the efforts and progress in the drug discovery research for agents targeting the 5-HT_6_ receptors. 

## 2. Methods

### 2.1. Literature Search

PubMed (United States National Library of Medicine, Bethesda, MD, USA) and clinical trial registries (ClinicalTrials.gov registry) were searched with terms including, but not limited to, “(serotonin-6 receptor) AND (Clinical trials)”, “(5-HT_6_ receptor) AND (Clinical trials)”, (5-HT_6_ receptor antagonist) AND (Clinical trials)”, with publication date restricted to between 1 January 1996 to 30 November 2022. The choice of this search date was based on the cloning of human 5-HT_6_ receptors in the year 1996. Additionally, searches were performed using internet-based search engines, such as Google and Google Scholar, to manually search for other relevant articles. 

The 5-HT_6_ receptor antagonists that were evaluated in patient populations (clinical trials) were considered and listed by code and compound name (if any). The literature on the non-clinical biology and clinical investigation of these 5-HT_6_ receptor antagonists were collected from PubMed or ClinicalTrials.gov registry or Google or Google scholar by searching with the term “(compound name) and (code)”, “(compound name) OR (code)”. 

### 2.2. Data Extraction

Information extracted from the clinical literature included indication, NCT number, endpoint and outcome. The information extracted from the non-clinical literature included the code(s), chemical structure, in-vitro profile, and in-vivo profile.

## 3. Results

**Figure 1:**
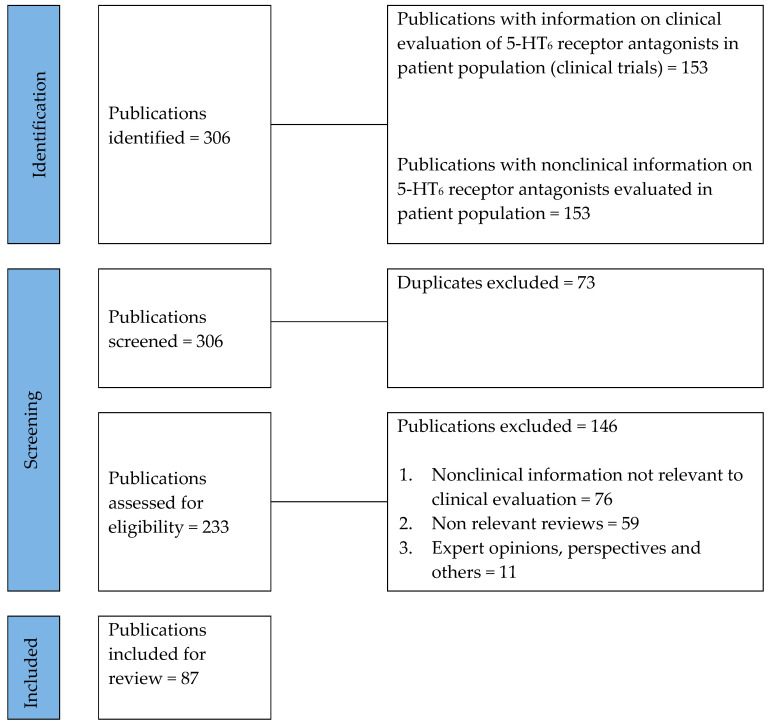
PRISMA flow diagram.

The searches resulted in 306 publications. Of these, 87 publications (reports, press releases or documents) were included in this review (Figure 1). Publication references are listed in Supplementary Material.

### 3.1. 5-HT_6_ Receptor and Drug Discovery

Initial interest in investigating the potential utility of 5-HT_6_ receptors for brain disorders arose from the studies suggesting that several clinically used psychotropics (e.g., olanzapine, clozapine, amitriptyline, nortriptyline, to name a few) functioned as 5-HT_6_ receptors antagonists [12,19]. Drug discovery efforts led to the identification of several selective 5-HT_6_ receptor agents that act as agonists or antagonists [20,21]. 5-HT_6_ receptor mediates its effects through other signaling pathways, including the mammalian target of rapamycin pathway [22,23,24,25], cyclin-dependent kinase [25,26,27], Fyn-tyrosine kinase [28], light chain 1 subunit of the microtubule-associated protein 1B [29], and Jun activation domain-binding protein-1 [30]. Research suggests that targeting specific downstream pathways (through functional selectivity) may have unique advantages and can assist in developing treatments with specific therapeutic value [31,32]. Although several 5-HT_6_ receptor agonists and antagonists with diverse physicochemical properties [20,33,34,35] and specific interaction modes with the receptors have been identified [20,26,27,33,34,35,36], such concepts are yet to be a basis for studies in patient populations.

**Figure 2:**
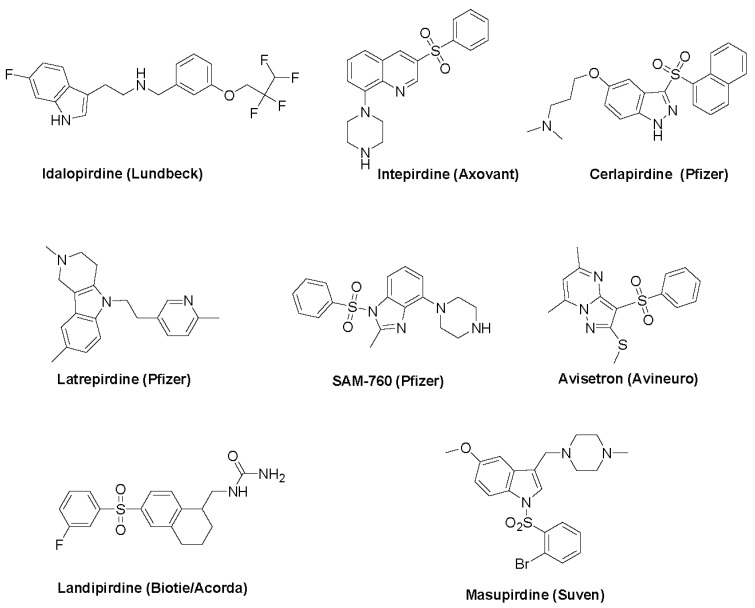
Clinical candidates targeting 5-HT_6_ receptors.

In animal models, both 5-HT_6_ receptor agonists and antagonists produced similar behavioral effects [37,38]. Most agents that were progressed to clinical evaluation were 5-HT_6_ receptor antagonists, and we focus on the therapeutic indications of this class of agents. The chemical structures of these agents are included in Figure 2 and their non-clinical and clinical profiles are included in Table 1, Table 2 and Table 3, respectively.

The initial interest of targeting schizophrenia through 5-HT_6_ receptors evolved from research indicating a potent affinity of several antipsychotics for the 5-HT_6_ receptors [8]. In addition, 5-HT_6_ receptor mRNA was found in the limbic system and striatum, brain regions implicated in the pathogenesis of schizophrenia [12]. A few 5-HT_6_ receptors antagonists have been studied in schizophrenia patients.

Subsequently, the focus from schizophrenia shifted to AD. Progressive cognitive impairment is the major disease symptom of AD and neuropsychiatric abnormalities are not uncommon [91]. Cholinergic hypofunction is a contributor to memory dysfunction [92]. Considering the involvement of 5-HT_6_ receptors in the modulation of cholinergic neurotransmission [93], 5-HT_6_ receptor antagonists were explored as potential treatments for cognitive impairment. Several 5-HT_6_ receptor antagonists were shown to enhance learning and memory in animal models (for reviews, see [16,42,94,95,96]). Most 5-HT_6_ receptor antagonists have been studied for potential cognitive benefits in AD patients based on the inferred cognitive benefits from animal models. 

Parkinson’s disease dementia (PDD) and dementia with Lewy bodies (DLB) are some of the other neurodegenerative disorders with dementia as a characteristic feature. Deficits/imbalance in the cholinergic neurotransmission has been thought to play a role in the dementias of PDD and DLB [97,98,99]. Considering the localization of 5-HT_6_ receptors in substantia nigra and cerebral cortex [12,18], and the modulatory role of 5-HT_6_ receptors on cholinergic neurotransmission, 5-HT_6_ receptor antagonists may have utility in the treatment of PDD or DLB. Few 5-HT_6_ receptor antagonists have been studied in PDD or DLB.

Neuropsychiatric symptoms (NPS) are heterogeneous non-cognitive behavioral or mood manifestations of neurodegenerative disorders like AD, PDD and DLB [100,101]. These symptoms include psychosis, agitation/aggression, depression, anxiety, euphoria, apathy, disinhibition, irritability, motor disturbance, night-time behavior, and appetite and eating changes [102,103]. NPS are associated with increased levels of caregiver burden and distress [104,105]. There is no approved treatment for the management of NPS, except pimavanserin for PD psychosis and risperidone for short-term use for behavioral and psychological symptoms of AD (in Europe, Canada, Australia and New Zealand). Considering the affinity of antipsychotics and antidepressants for 5-HT_6_ receptors and the wide distribution of 5-HT_6_ receptors in the brain regions implicated for the control of mood and behavior, 5-HT_6_ agents are candidates for use in the treatment of NPS of AD. 

The following section describes the profile of the 5-HT**_6_** receptor antagonists in patient populations. 

### 3.2. Avisetron

In addition to affinity for 5-HT_6_ receptors, avisetron has affinity for 5-HT_2B_ receptors [39]. In an animal model, avisetron attenuated the effects of apomorphine on the startle prepulse inhibition, suggesting potential effects on the positive symptoms of schizophrenia. Avisetron also attenuated the memory deficits induced by MK-801 in task involving object recognition, passive avoidance, and route memory in the Morris water maze task [39,68] indicating potential effects on the cognitive symptoms of schizophrenia. 

Initially the effect of avisetron (4 mg, every day (QD)) was studied in schizophrenia patients receiving stable antipsychotic medication. After treatment for 28 days, those treated with avisetron showed improvement in the PANSS total scores compared to the baseline, whereas no changes were observed with placebo treatment. In addition, significant decreases in PANSS positive subscores were observed with avisetron treatment. Significant changes were also noted in the continuous attention task. Overall, the results were suggestive of beneficial cognitive and anti-psychotic effects of avisetron [69,70]. 

Subsequently, avisetron was studied for its effects in schizophrenia patients with incomplete remission after receiving stable doses of antipsychotics. The starting dose of avisetron was 4 mg (QD) and was increased to 8 mg (QD) after 1 week. Avisetron treatment showed a nonsignificant trend towards improvement over placebo in PANSS total scores, PANSS positive subscores, and PANSS general psychopathology scale. Overall, avisetron was safe and well-tolerated [70]; however, no further evaluation of avisetron for schizophrenia is reported. 

### 3.3. Cerlapirdine

This agent is a potent 5-HT_6_ receptor antagonist with an affinity for 5-HT_7_ and 5-HT_2B_ receptors. Cerlapirdine attenuated scopolamine- and MK-801-induced deficits in the object recognition and contextual fear conditioning tasks. It also attenuated the combined scopolamine and MK-801-induced deficit in the object recognition task. Cerlapirdine was shown to modulate acetylcholine and glutamate in the hippocampus [40].

Based on the pharmacological profile, cerlapirdine (0.5 mg, 1.5 mg, 3 mg and 5 mg, QD) was evaluated in an exploratory study as a monotherapy in mild to moderate AD patients for treatment duration of 4 weeks. Although the primary objective was to assess the safety and tolerability, the efficacy was explored using the MMSE, ADAS-Cog, and subtests of the Cambridge Neuropsychological Test Automated Battery (CANTAB). Trends towards improvement favoring the cerlapirdine treatment were observed on the ADAS-Cog scores and CANTAB [71].

Subsequently, cerlapirdine (1.5 mg, 3 mg and 5 mg, QD) was evaluated in a 52-week phase 2 study for its effects on cognition in mild to moderate AD patients. The primary outcome measure was change from baseline in ADAS-Cog scores at week 24. The study was terminated for reasons of futility (NCT00895895) [72]. Futility may be attributed to low 5-HT_6_ receptor occupancy i.e., <30% after multiple 5 mg doses [106]. No safety concerns were noted with cerlapirdine in the clinical studies.

### 3.4. Idalopirdine

In addition to potently blocking the 5-HT_6_ receptors, idalopirdine has potent affinity for adrenergic receptors (α_1A_ and α_1B_) and moderate affinity for 5-HT_2A_ and 5-HT_2C_ receptors [41]. In the conditioned avoidance response task, idalopirdine potentiated the effects of haloperidol and risperidone [54] suggesting its potential as an adjunct treatment for schizophrenia. In a rat model, idalopirdine as a standalone treatment attenuated the subchronic phencyclidine-induced cognitive impairment, suggesting its potential utility in the treatment of cognitive impairment associated with schizophrenia [41]. 

In clinical studies, idalopirdine was evaluated as monotherapy in a small population of 20 schizophrenic patients treated with a dose-escalating regimen of idalopirdine (60/180 mg or 120/240 mg) or placebo. The treatment duration was 14 days, and frequency was once a day. The Brief Assessment of Cognition in Schizophrenia (BACS) was used to assess the changes in cognition. A dose-dependent improvement was observed on BACS, with the effect reaching statistical significance at the highest tested dose of 240 mg. No changes were observed in the placebo treatment arm [73].

Further, idalopirdine was evaluated as an augmentation therapy to risperidone (NCT00810667). Idalopirdine (60 mg, BID) did not show improvement over placebo in schizophrenia symptoms as assessed by Positive and Negative Syndrome Scale (PANSS) total scores at the end of 12 weeks’ treatment. In addition, there were no changes in the BACS scores or the PANSS cognitive subscale scores [74]. Overall, idalopirdine was safe and well-tolerated in schizophrenic patients. No further evaluation of idalopirdine in schizophrenia population has been reported. 

Subsequently, idalopirdine was repositioned for the treatment of AD. In animal models, no procognitive-like effects of idalopirdine were observed as a standalone treatment. However, idalopirdine potentiated the effects of donepezil on neuronal oscillations, extracellular brain acetylcholine levels, and blood oxygen level-dependent functional signaling [55,56,57].

Guided by observations in animal models, idalopirdine (30 mg, three times a day (TID)) was studied for its effects on cognition in moderate AD patients as an add-on therapy to donepezil in a phase 2 study. The effect on cognition was assessed based on the change from baseline in ADAS-Cog 11 scores at week 24. A significant improvement in ADAS-Cog 11 scores was observed in the idalopirdine treatment arm compared to placebo. Parallel improvement was also observed in the MMSE scale. A trend towards improvement was observed in functional (ADCS-ADL) and global (Alzheimer’s Disease Cooperative Study Clinical Global Impression of Change (ADCS-CGIC)) outcome measures [75]. Post hoc analysis of the phase 2 study suggested that treatment was associated with improvements in the anxiety and hallucinations domains of the 12-item Neuropsychiatric Inventory scale (NPI-12) [107].

Based on the effects on cognition noted in the phase 2 trial, idalopirdine was evaluated in phase 3 studies as an add-on therapy to donepezil or other cholinesterase inhibitors. The phase 3 studies differed from the phase 2 study, in dosage (10 mg or 30 mg or 60 mg for phase 3 vs. 30 mg for phase 2), and frequency of the idalopirdine treatment (QD for phase 3 vs. TID for phase 2), background therapy (all cholinesterase inhibitors for phase 3 vs. only donepezil for phase 2), cognitive impairment severity (MMSE12-22, mild to moderate for phase 3 vs. MMSE12-19, moderate for phase 2), and study geography (worldwide for phase 3 vs. Europe, Australia, Czech Republic and Canada for phase 2). No effects of idalopirdine were observed on ADAS-Cog 11 scores or other secondary endpoints compared to placebo [76]. In phase 3 studies, the effect of idalopirdine on anxiety was followed-up by selectively studying the anxiety domain of the NPI-12 scale as one of the secondary endpoints. No notable effect was observed (NCT02006641). Overall, idalopirdine was found to be safe and well-tolerated in the clinical studies.

### 3.5. Intepirdine 

This agent has affinity for 5-HT_6_ and 5-HT_2A_ receptors [42,108]. In animal models, intepirdine enhanced cholinergic neurotransmission, induced neural plasticity, and enhanced cognition [58]. Intepirdine potentiated the effects of donepezil on cholinergic neurotransmission [59].

In AD patients with mild to moderate cognitive deficits, intepirdine (5 mg, 15 mg and 35 mg, QD) was evaluated as a monotherapy for 24 weeks [77]. The effects on global function and cognition were assessed based on the Clinician’s Interview-Based Impression of Change with caregiver input (CIBIC+) and change from baseline in Alzheimer’s Disease Assessment Scale-Cognitive subscale (ADAS-Cog) scores, respectively. Intepirdine treatment over 24 weeks showed improvement in CIBIC+, and the effect reached nominal statistical significance over placebo. No treatment-related effects were observed on the change from baseline in ADAS-Cog scores; however, slope of the dose response from linear trend analysis showed an improvement. Subsequent post hoc analysis suggested beneficial effects of intepirdine on the ADAS-Cog scores in a subgroup of the population with a Mini-Mental State Examination (MMSE) score of <18. 

Two trials evaluated the effects of contemporary treatment with intepirdine and donepezil for 24 weeks in mild to moderate AD patients [78,79]. Intepirdine was evaluated at doses of 15 mg and 35 mg (QD) in one study, and another study titrated intepirdine from 15 mg (QD) to 35 mg (QD) at week 4. The co-primary endpoints were change from baseline in CIBIC+ and ADAS-Cog scores. No significant effects of either intepirdine or donepezil were observed on either of the endpoints. The effects of intepirdine were consistent between studies. 

Intepirdine (15 mg and 35 mg, QD) was also studied as an add-on therapy to donepezil for 48 weeks in mild to moderate AD patients [79]. The primary endpoints were Clinical Dementia Rating-Sum of Boxes (CDR-SB) and change from baseline on the ADAS-Cog scores at week 24. Treatment with intepirdine was associated with significant improvement in ADAS-Cog scores at week 24 over placebo, and the effects persisted for up to 48 weeks. Parallel improvements were observed in the function as assessed by Alzheimer’s Disease Cooperative Study-Activities of Daily Living (ADCS-ADL). 

The observation of potential beneficial effects in earlier studies led to a large phase 3 study evaluating intepirdine (35 mg, QD) as an add-on treatment to donepezil for 24 weeks [80]. The change from baseline in ADAS-Cog scores and ADCS-ADL were co-primary endpoints. No notable effect of intepirdine treatment was observed on either of the endpoints. Intepirdine treatment was associated with a favorable safety profile across all the studies.

The effects of intepirdine (35 mg and 70 mg, QD) were assessed in patients with DLB. The primary efficacy measure was assessment of the effects of intepirdine on motor functions as change from baseline in the Unified Parkinson’s Disease Rating Scale–Part III (UPDRS–III) scores at week 24. Secondary outcome measures assessed the effects on cognition as change from baseline in the ADAS-Cog scores. No significant effects of intepirdine were observed in the change from baseline in the UPDRS–III or ADAS-Cog 11 scores compared to placebo. Intepirdine was well-tolerated, with higher incidences of gastrointestinal adverse events [81].

### 3.6. Landipirdine

Landipirdine is a potent blocker of both 5-HT_6_ and 5-HT_2A_ receptors. No reports on the non-clinical profile of landipirdine are publicly available. Landipirdine was evaluated for its effect on cognition in PDD patients as an add-on therapy to cholinesterase inhibitor for 16 weeks. The dose of landipirdine was up-titrated: 20 mg (QD) for week 1, 50 mg (QD) for week 2, and 100 mg (QD) from week 3 through week 16. The effect on cognition was assessed as change from baseline in Cognitive Drug Research Computerized Drug Research Cognition Battery Continuity of Attention (CDRCOA) captured in the ON state. No effects were observed on change from baseline in CDRCOA compared to placebo. Worsening of motor symptoms was observed in those receiving landipirdine [82]. In a post hoc analysis, examination of NPS based on the NPI-12 scale suggested potential improvements in apathy, anxiety, and irritability/lability domains with landipirdine treatment over placebo [109]. No further development of landipirdine has been reported.

### 3.7. Latrepirdine

This 5-HT_6_ receptor antagonist has affinities for acetylcholinesterases, N-methyl-D-aspartate receptors, and voltage-gated calcium channels. Many of the effects of latrepirdine were believed to be mediated through the blockade of 5-HT_6_ receptors [43]. Based on the affinity for 5-HT_6_ receptors, latrepirdine (20 mg) was studied as an add-on to risperidone therapy in schizophrenic patients transitioning from an acute psychotic episode to symptom remission [83]. No notable differences were observed in the PANSS total or subscale scores between the latrepirdine and placebo treated groups. However, latrepirdine showed statistically significant improvement over the placebo in the 16-item Negative Symptom Assessment (NSA-16) and numerical improvement in cognitive dimensions (working memory, attention, psycho-motor coordination and planning) in comparison with the placebo-treated group. No further studies for latrepirdine in schizophrenic patients have been reported.

In animal models, latrepirdine treatment was associated with memory improvement in a social recognition task [43] and an object recognition task [63]. The interest of developing latrepirdine for AD was based on the outcome from an 8-week open label pilot study. Latrepirdine was evaluated at a dose of 20 mg, TID. The study found improvements in cognition, function, and NPS [44]. An ensuing proof-of-concept study reported similar observations on cognition (assessed based on change from baseline in ADAS-Cog), function (assessed based on change from baseline in ADCS-ADL) and neuropsychiatric symptoms (assessed based on change from baseline in NPI-12). Latrepirdine was evaluated at a dose of 20 mg, TID after 26 weeks of treatment [84]. The effect of latrepirdine on the individual NPS was not reported, except for an absence of effects on dysphoria/depression [44,84]. In the subsequent larger phase 3 studies that were conducted to replicate the earlier findings, no effect was observed on cognition or functions as a standalone treatment for 26 weeks (NCT00675623) or as an add-on treatment to donepezil for 52 weeks (NCT00829374). In the above studies, latrepirdine was evaluated at doses of 5 mg and 20 mg, TID [85]. The effects on NPS were not reported in the results from the phase 3 studies. Latrepirdine was safe and well-tolerated in AD patients.

### 3.8. Masupirdine

This therapeutic candidate is a potent 5-HT_6_ receptor antagonist and lacks affinity for other serotonergic receptors subtypes at clinically relevant doses. Masupirdine showed procognitive effects in diverse animal models as standalone and add-on treatment to donepezil. Masupirdine showed beneficial effects on cognition in animals receiving treatment with donepezil and memantine [49,65]. In animal models, masupirdine significantly reduced aggression-like behaviors in the resident–intruder task and decreased dominance levels in the dominant–submissive assay. In addition, masupirdine modulated the cortical dopamine and norepinephrine levels assessed using the brain microdialysis in rats [66].

Contingent on the procognitive effects of masupirdine in combination with donepezil and memantine, a phase 2 study evaluated masupirdine (50 mg and 100 mg, QD) as an add-on treatment to donepezil and memantine in moderate AD patients. The primary efficacy endpoint was the change from baseline in ADAS-Cog scores after 26 weeks of treatment. No notable effects of masupirdine were observed on cognition when compared to the placebo treatment. Overall, masupirdine was safe and well-tolerated in AD patients [86]. A post hoc analysis revealed that masupirdine may have cognitive benefits in patients who are not concurrently treated with memantine high dose (28 mg) [87]. Post hoc observations from the phase 2 study suggested that treatment with masupirdine was associated with improvements in domains of agitation/aggression and psychosis [88].

### 3.9. SAM-760

It is a potent 5-HT_6_ receptor antagonist. SAM-760 also acts as an antagonist at 5-HT_2A_ receptors; however, it did not show any significant occupancy of cortical 5-HT_2A_ receptors in humans [110]. SAM-760 showed procognitive effects in diverse animal models including cholinergic and glutamatergic deficit models. It enhanced the cholinergic and glutamatergic neurotransmission in the rat hippocampus and prefrontal cortex [50,51]. 

SAM-760 was advanced for clinical evaluation to assess its effect on cognition in moderate AD patients with existing NPS. Twelve weeks of treatment with SAM-760 (30 mg, QD) produced no significant effects in the change from baseline in ADAS-Cog scores. No beneficial effects were observed in the secondary outcome measures including NPS as assessed by the NPI-12 scale. Overall, SAM-760 was safe and well-tolerated [90].

## 4. Conclusions and Outlook

5-HT_6_ receptors have received considerable attention as potential treatments for cognitive deficits or NPS associated with AD and other neurocognitive disorders. Blocking the functions of 5-HT_6_ receptors has been the biological effect of agents evaluated in clinical trials. Although the initial focus of 5-HT_6_ receptors research was intended for the treatment of schizophrenia, most of the advanced clinical studies focus on memory deficits associated with AD or other dementias. However, no conclusive evidence has been observed in clinical trials to support the utility of targeting 5-HT_6_ receptor antagonists for the treatment of schizophrenia or memory deficits in dementias. The majority of these agents had affinity for other (non-5-HT_6_) serotonergic receptors, which might partially explain their failure(s).

5-HT_6_ receptor agents have been shown to have anxiolytic and antidepressant-like properties in animal models [37]. 5-HT_6_ receptor antagonists also facilitate release of neurotransmitters, such as dopamine and norepinephrine, implicated in mood and behavior [42,59,111]. Based on the affinity of many psychotropic drugs for 5-HT_6_ receptors, the observations of modulatory role of the receptor on mood, and potential beneficial effects on NPS in patients with dementia, 5-HT_6_ receptors antagonists may have potential as treatments of NPS in dementia. Many of the 5-HT_6_ receptor antagonists also blocked the 5-HT_2A_ receptors. Considering the beneficial effects of 5-HT_2A_ receptors blockade in psychiatry, it is essential to understand the role of 5-HT_6_ receptor on NPS. Characterizing the clinically evaluated agents for their interaction modes with the receptor may further help to delineate agents, and selective 5-HT_6_ receptor antagonist may target specific downstream pathways and avoid unwanted effects. Among the 5-HT_6_ receptor antagonists, masupirdine is being evaluated for its effects on agitation in patients with Alzheimer’s-type dementia in a potentially pivotal clinical trial (NCT05397639). The outcome of 5-HT_6_ receptor antagonists in the past clinical trials has been disappointing. The current trial of masupirdine for agitation will inform future approaches to the clinical utility of selective 5-HT_6_ receptor antagonists. 

## Figures and Tables

**Table 1:** In-vitro Profile of 5-HT_6_ Receptor Antagonists in Clinical Development.

Agents	In-Vitro Profile		
	5-HT_6_ Receptors	Other Serotonergic Receptors	Other Receptors
**Avisetron** (AVN-211)	K_i_ 1.09 nM K_b_ 0.83–1.97 nM [39]	5-HT_1A_ IC_50_ > 1000 nM; 5-HT_1B_ IC_50_ > 1000 nM; 5-HT_2A_ IC_50_ > 1000 nM; 5-HT_2B_ IC_50_ 96 nM; 5-HT_2C_ IC_50_ > 1000 nM; 5-HT_3_ IC_50_ > 1000 nM; 5-HT_4_ IC_50_ > 1000 nM; 5-HT_7_ IC_50_ >1000 nM [39]	5000-fold selectivity over 65 other receptors, enzymes, and ion channels [39]
**Cerlapirdine** (SAM-531 or WAY-262531)	Ki 1.3 nM [40]	5-HT_2B_ K_i_ 440 nM; 5-HT_7_ K_i_ 881 nM; 5-HT_1B_ IC_50_ > 1000 nM; 5-HT_1D_ IC_50_ > 1000 nM; 5-HT_2A_ IC_50_ > 1000 nM; 5-HT_2C_ IC_50_ > 1000 nM [40]	Not reported
**Idalopirdine** (LY 483518 or SGS518 or Lu AE58054)	Ki 0.83 nM; EC_50_ 25 nM; K_b_ 4.9 nM [41]	5-HT_1A_ K_i_ 2300 nM; 5-HT_1B_ K_i_ > 10,000 nM; 5-HT_1D_ K_i_ 2600 nM; 5-HT_1E_ K_i_ > 4600 nM; 5-HT_1F_ K_i_ 2400 nM; 5-HT_2A_ K_i_ 83 nM; 5-HT_2B_ K_i_ > 4100 nM; 5-HT_2C_ K_i_ 250 nM; 5-HT_3_ IC_50_ > 10,000 nM; 5-HT_4e_ IC_50_ > 10,000 nM; 5-HT_7_ K_i_ > 10,000 nM [41]	>50-fold selectivity over 100 other receptors, enzymes, and ion channels, except for adrenergic receptors (α_1A_ Ki 21 nM; α_1B_ Ki 22 nM) [41]
**Intepirdine** (SB-742457 or RVT-101)	K_i_ 0.23 nM [42]	5-HT_2A_ K_i_ 10 nM [42]	>100-fold selectivity over other receptors, enzymes, and ion channels [42]
**Landipirdine** (SYN-120)	Not reported		
**Latrepirdine** (Dimebon)	K_i_ 26 nM; K_b_ 26 nM [43]	Not reported	Weak inhibitor of cholinesterase, N-methyl-D-aspartate and voltage-gated calcium channels and weak modulator of the mitochondrial permeability transition pore [44,45,46,47,48]
**Masupirdine** (SUVN-502)	K_i_ 2.04 nM K_b_ 2.6 nM [49]	5-HT_1A_ K_i_ 7020 nM; 5-HT_1B_ IC_50_ > 10,000 nM; 5-HT_1D_ IC_50_ > 10,000 nM; 5-HT_2A_ K_i_ 2514 nM; 5-HT_2C_ K_i_ > 1000 nM; 5-HT_4B_ K_i_ 4166 nM; 5-HT_5A_ IC_50_ > 10,000 nM; 5-HT_7_ IC_50_ > 10,000 nM [49]	>500-fold selectivity over 100 other targets that includes receptors, ion channels, enzymes, peptides, growth factors, steroids, immunological factors, second messengers, and prostaglandins except for dopamine receptor (D_3_ K_i_ 616 nM) and adrenergic receptors (α_2A_ K_i_ 2570 nM; α_2C_ K_b_ 619 nM) [49]
**SAM-760** (WYE-103760 or PF-05212377)	K_i_ 0.53 nM; IC_50_ 0.76 nM [50,51]	Not reported	

**Table 2:** Metabolic and In-vivo Profile of 5-HT_6_ Receptor Antagonists Recently in Development.

Agents	Oral Bioavailability (%)	CYP Isoform Involved in the Metabolism	Active Metabolite	Drug Interaction Liability	In-Vivo Profile
**Avisetron**	5.73 [39]	Not reported		Inhibitor of CYP 2B6, 2C9, 2C19 [39]	Attenuated the memory deficits induced by MK-801, and scopolamine in the object recognition task, passive avoidance task, and Morris water maze task [39]
**Cerlapirdine**	24 [40]	Not reported		None [40]	Attenuated the memory deficits induced by MK-801, scopolamine, combined scopolamine and MK-801 treatment in the object recognition task [40]
**Idalopirdine**	60 ^#^ [52]	CYP3A4 and CYP2D6 [53]	Not reported	Low [52]	Improved cognition in phencyclidine-challenged rats; modulated dopamine, norepinephrine and glutamate neurotransmitters in brain; potentiated the effects of donepezil on neuronal oscillations, acetylcholine modulation and blood oxygen level-dependent functional signaling [54,55,56,57]
**Intepirdine**	76 [42]	Not reported			Attenuated the memory deficits caused by scopolamine in the object recognition task and passive avoidance task and reversed the memory deficit associated with senile dementia; enhanced medial prefrontal cortex cholinergic neurotransmission as standalone and add-on treatment to donepezil [58,59]
**Landipirdine**	Not reported				
**Latrepirdine**	53 [60]; 5 to 6% in extensive CYP2D6 metabolizers and 45% in poor CYP2D6 metabolizers * [61]	CYP2D6 [62]	Not reported		Enhanced memory in the social recognition task and object recognition task [43,63]
**Masupirdine**	24.9 [49]	CYP3A4 [64]	Yes [64]	No ^$^ [64]	Attenuated scopolamine, MK-801 and ageing associated memory deficits; potentiated the effects of donepezil on neuronal oscillations, and acetylcholine modulation; potentiated the effects of memantine on acetylcholine modulation; potentiated the effects of donepezil and memantine on cognition in the Morris water maze task, neuronal oscillations, and acetylcholine modulation; reduced aggression-like behaviors in resident intruder task and dominance levels in the dominant–submissive assay; Modulated cortical dopamine and norepinephrine [65,66]
**SAM-760**	Not reported	CYP3A4/5 [67]	Not reported	No [67]	Attenuated the memory deficits induced by MK-801, scopolamine, combined scopolamine and MK-801 treatment in the object recognition task [50,51]

^#^ Absolute bioavailability of the idalopirdine immediate release tablet in healthy male subjects; * Estimated oral bioavailability in extensive and poor CYP2D6 metabolizers; ^$^ Based on in-vitro CYP profile.

**Table 3:** Clinical Profile of 5-HT_6_ Antagonists Recently in Development.

Agents	Indication	NCT Number	Endpoint	Outcome
**Avisetron**	Schizophrenia (Pilot study) [68,69]	Not available	**Key Endpoints:** PANSS, CAT	As augmentation therapy, avisetron treatment-related benefits were observed in PANSS total scores, PANSS positive subscores and CAT
	Schizophrenia (Phase 2 study) [70]	Not available	**Primary:** Change from baseline in the PANSS total scores **Other Key Endpoints:** Change from baseline in the CGI-S, CGI-I, NSA-16, PSPS, CogFu, BACS and CPT	As augmentation therapy, a trend towards avisetron treatment-related benefits were observed in PANSS total score, PANSS positive subscores and PANSS general psychopathology scale after 6 weeks of treatment; No notable effects on CGI-S, CGI-I, NSA, PSPS, CogFu, BACS and CPT
**Cerlapirdine**	Alzheimer’s disease (Pilot study) [71]	NCT00481520	**Key Endpoints:** MMSE, ADAS-Cog and CANTAB	Trend towards improvement was observed with cerlapirdine treatment on the ADAS-Cog 11 and CANTAB at the end of 4 weeks
	Alzheimer’s disease (Phase 2 study) [72]	NCT00895895	**Primary:** Change from baseline in the ADAS-Cog 11 total scores **Other Key Endpoints:** Change from baseline in the ADCS-CGIC, CANTAB and NPI-12	No beneficial effects of cerlapirdine were observed at the end of 24 weeks on any of the studied endpoints
**Idalopirdine**	Schizophrenia (Pilot study) [73]	Not available	Safety, tolerability, pharmacokinetics and pharmacodynamics (cognitive changes assessed using BACS)	Safe and well-tolerated as standalone treatment for 14 days; Idalopirdine treatment was associated with dose-dependent pattern of improvement in the BACS endpoint; No effect in the placebo treated group
	Schizophrenia (Phase 2 study) [74]	NCT00810667	**Primary:** Change in PANSS total scores **Other Key Endpoints:** Neurocognitive performance using the BACS	As augmentation therapy, no change was observed in the PANSS total scores or BACS scores or PANSS cognitive subscale scores as compared to placebo after 12 weeks of treatment
	Alzheimer’s disease (Phase 2 study) [75]	NCT01019421	**Primary:** Change from baseline in the ADAS-Cog 11 **Other Key Endpoints:** Change from baseline in the ADCS-ADL, ADCS-CGIC, MMSE and NPI-12	As augmentation therapy, significant improvements in ADAS-Cog 11 scores were observed as compared to placebo after 24 weeks of treatment; Parallel trend towards improvement in ADCS-ADL and ADCS-CGIC; Improvements in anxiety and hallucinations domains of the NPI-12 scale in a post hoc analysis
	Alzheimer’s disease (Phase 3 studies) [76]	NCT01955161, NCT02006641, and NCT02006654	**Primary:** Change from baseline in the ADAS-Cog **Other Key Endpoints:** Change from baseline in the ADCS-ADL, ADCS-CGIC, MMSE and NPI-12	As augmentation therapy, no significant improvements in ADAS-Cog 11 scores as compared to placebo after 24 weeks of treatment; Similar observations were noted in other endpoints
**Intepirdine**	Alzheimer’s disease (Phase 2 study) [77]	NCT00224497	**Primary:** Change from baseline in the ADAS-Cog 11 scores and CIBIC+ **Other Key Endpoints:** Change from baseline in the MMSE and NPI-12	Significant improvement in the CIBIC+ and trend in the ADAS-Cog 11 scores was observed with 24 weeks of intepirdine treatment
	Alzheimer’s disease (Phase 2 studies) [78,79]	NCT00348192 and NCT00708552	**Primary:** Change from baseline in the ADAS-Cog 11 scores and CIBIC+ **Other Key Endpoints:** Change from baseline in the MMSE and ADCS-ADL	No significant effect was observed on the CIBIC+ or ADAS-Cog 11 scores at the end of 24 weeks of intepirdine treatment
	Alzheimer’s disease (Phase 2 study) [79]	NCT00710684	**Primary:** Change from baseline in the ADAS-Cog 11 scores and CDR-SB **Other Key Endpoints:** Change from baseline in the MMSE and ADCS-ADL	As an add-on therapy to donepezil, beneficial effects of intepirdine were observed in the ADAS-Cog 11 scores at the end of 24 weeks and the effects were noted up to 48 weeks; No notable effects were observed on the CDR-SB
	Alzheimer’s disease (Phase 3 study) [80]	NCT02585934	**Primary:** Change from baseline in the ADAS-Cog 11 scores and ADCS-ADL **Other Key Endpoints:** Change from baseline in the NPI-12	As an add-on therapy to donepezil, no beneficial effects of intepirdine were observed in the ADAS-Cog 11 or ADCS-ADL scores at the end of 24 weeks
	Dementia with Lewy bodies (Phase 2 study) [81]	NCT02669433	**Primary:** Change from baseline in the UPDRS–III total scores **Other Key Endpoints:** Change from baseline in the ADAS-Cog 11 and CIBIC+	No beneficial effects of intepirdine were observed in the UPDRS–III total score at the end of 24 weeks
**Landipirdine**	Parkinson’s disease dementia (Phase 2 study) [82]	NCT02258152	**Primary:** Change from baseline in the CDRCOA total scores **Other Key Endpoints:** Change from baseline in the ADCS-CGIC, MoCA and NPI-12	No beneficial effects of landipirdine were observed as an add-on treatment to cholinesterase inhibitor after 16 weeks of treatment; Post hoc analysis suggested beneficial effects on apathy, anxiety, and irritability/lability
**Latrepirdine**	Schizophrenia (Phase 2 study) [83]	Not available	**Key Endpoints:** PANSS, CGI-S and NSA-16	As an add-on therapy, no beneficial effect of latrepirdine was observed in the PANSS total or sub scale scores; Latrepirdine showed statistically significant improvement in the NSA-16 scale
	Alzheimer’s disease (Pilot study) [44]	Not available	**Key Endpoint:** Bukatina scale	Treatment with latrepirdine was associated with improvements in cognitive function and reduction of NPS
	Alzheimer’s disease (Phase 2 study) [84]	NCT00377715	**Primary:** Change from baseline in the ADAS-Cog 11 scores **Other Key Endpoints:** Change from baseline in the MMSE, ADCS-ADL and NPI-12	Significant improvement was observed on the ADAS-Cog 11, MMSE, ADCS-ADL and NPI-12 after 24 weeks of treatment
	Alzheimer’s disease (Phase 3 studies) [85]	NCT00675623 and NCT00829374	**Primary:** Change from baseline in the ADAS-Cog 11 scores and CIBIC+ (NCT00675623) or change from baseline in the ADAS-Cog 11 scores and ADCS-ADL (NCT00829374) **Other Key Endpoints:** Change from baseline in the MMSE, ADCS-ADL and NPI-12	No significant effect of latrepirdine treatment was observed as standalone or add-on to donepezil after 26 or 52 weeks of treatment
**Masupirdine**	Alzheimer’s disease (Phase 2 study) [86,87,88]	NCT02580305	**Primary:** Change from baseline in the ADAS-Cog 11 total scores **Other Key Endpoints:** Change from baseline in the ADCS-ADL, MMSE, CDR-SB and NPI-12	No beneficial effects of masupirdine were observed as an add-on treatment to donepezil and memantine in the ADAS-Cog 11 after 26 weeks of treatment; Post hoc analysis suggested potential impact of memantine on the efficacy, and potential beneficial effects on agitation/aggression and psychosis
	Alzheimer’s disease Agitation (Potentially pivotal study) [89]	NCT05397639	**Primary:** Change from baseline in the CMAI items scores aligning to the International Psychogeriatric Association agitation criteria domains **Other Key Endpoints:** Change from baseline in the modified ADCS-CGI-C, MMSE and ADAS-Cog 11	Study in progress
**SAM-760**	Alzheimer’s disease (Phase 2 study) [90]	NCT01712074	**Primary:** Change from baseline in the ADAS-Cog 13 total scores **Other Key Endpoints:** Change from baseline in the COWAT, CFT and NPI-12	Trial was stopped after a futility analysis; No beneficial effect of SAM-760 treatment was observed after 12 weeks of treatment

ADAS-Cog—Alzheimer’s Disease Assessment Scale-Cognitive subscale; ADAS-Cog 11—Alzheimer’s Disease Assessment Scale-Cognitive subscale 11; ADAS-Cog 13—Alzheimer’s Disease Assessment Scale-Cognitive subscale 13; ADCS-ADL—Alzheimer’s Disease Cooperative Study—Activities of Daily Living; ADCS-CGIC—Alzheimer’s Disease Cooperative Study Clinical Global Impression of Change; BACS—Brief Assessment of Cognition in Schizophrenia; CANTAB—Cambridge Neuropsychological Test Automated Battery; CAT—Continuous Attention Task; CDRCOA—Computerized Drug Research Cognition Battery Continuity of Attention; CDR-SB—Clinical Dementia Rating scale Sum of Boxes; CFT—Category Fluency Test; CGI-I—Clinical Global Impression–Improvement; CGI-S—Clinical Global Impression–Severity; CIBIC+—Clinician’s Interview-Based Impression of Change with caregiver input; CMAI—Cohen-Mansfield Agitation Inventory; CogFu—Scale for Rating Functioning Related to Cognitive Impairment in Schizophrenia; COWAT—Controlled Oral Word Association Test; CPT—Continuous Performance Test; MMSE—Mini-Mental State Examination; MoCA—Montreal Cognitive Assessment. NPI-12—12-item Neuropsychiatric Inventory; NPS—Neuropsychiatric symptoms; NSA-16—16-item Negative Symptom Assessment; PANSS—Positive and Negative Syndrome Scale; PSPS—Personal and Social Performance Scale; UPDRS–III—Unified Parkinson’s Disease Rating Scale–Part III.

## Data Availability

The data presented in this study is available in the Supplementary Materials.

## Supplementary Materials

The following supporting information can be downloaded at: https://www.mdpi.com/article/10.3390/biom13020309/s1, Publication references.
