# Supplementary material for: Progress in Investigational Agents Targeting Serotonin-6 Receptors for the Treatment of Brain Disorders
Source: Biomolecules. 2023 Feb 7;13(2):309. doi: 10.3390/biom13020309 (PMC9953539; doi:10.3390/biom13020309)
Supplement: Supplementary file 1 [file biomolecules-13-00309-s001.zip › biomolecules-2157144-supplementary.pdf]

1. Monsma, F.J Jr.; Shen, Y.; Ward, R.P.; Hamblin, M.W.; Sibley, D.R. Cloning and expression of a novel serotonin receptor with high affinity for tricyclic psychotropic drugs. *Mol Pharmacol.* **1993**, 43(3), 320-327.
2. Kohen, R.; Metcalf, M.A.; Khan, N.; Druck T.; Huebner, K.; Lachowicz, J.E.; Meltzer, H.Y.; Sibley, D.R.; Roth, B.L.; Hamblin, M.W. Cloning, characterization, and chromosomal localization of a human 5-HT6 serotonin receptor. *J Neurochem*. **1996**, 66(1), 47-56.
3. Codony, X.; Burgueño, J.; Ramírez, M.J.; Vela, J.M. 5-HT6 receptor signal transduction second messenger systems. *Int Rev Neurobiol*. **2010**, 94, 89-110.
4. Riccioni, T. 5-HT6 receptor characterization. *Int Rev Neurobiol.* **2010**, 94, 67-88.
5. Woolley, M.L.; Marsden, C.A.; Fone, K.C. 5-ht6 receptors. Curr Drug Targets *CNS Neurol Disord.* **2004**, 3(1), 59-79.
6. Brodsky, M.; Lesiak, A.J.; Croicu, A.; Cohenca, N.; Sullivan, .JM.; Neumaier J.F. 5-HT6 receptor blockade regulates primary cilia morphology in striatal neurons. *Brain Res.* **2017**, 1660, 10-19.
7. Gérard, C.; Martres, M.P.; Lefèvre, K.; Miquel, M.C.; Vergé, D.; Lanfumey, L.; Doucet, E.; Hamon, M.; el Mestikawy, S. Immuno-localization of serotonin 5-HT6 receptor-like material in the rat central nervous system. Brain Res. 1997, 746(1-2), 207-19.
8. Roth, B.L.; Craigo, S.C.; Choudhary, M.S.; Uluer, A.; Monsma, F.J Jr.; Shen, Y.; Meltzer, H.Y.; Sibley, D.R. Binding of typical and atypical antipsychotic agents to 5-hydroxytryptamine-6 and 5-hydroxytryptamine-7 receptors. J Pharmacol Exp Ther. 1994, 268(3), 1403-1410.
9. Bali, A.; Singh, S. Serotonergic 5-HT6 Receptor Antagonists: Heterocyclic Chemistry and Potential Therapeutic Significance. Curr Top Med Chem. 2015, 15(17), 1643-1662.
10. Karila, D.; Freret, T.; Bouet, V.; Boulouard, M.; Dallemagne, P.; Rochais, C. Therapeutic Potential of 5-HT6 Receptor Agonists. J Med Chem. 2015, 58(20), 7901-7912.
11. Meffre, J.; Chaumont-Dubel, S.; Mannoury la Cour, C.; Loiseau, F.; Watson, D.J.; Dekeyne, A.; Séveno, M.; Rivet, J.M.; Gaven, F.; Déléris, P.; Hervé, D.; Fone, K.C.; Bockaert, J.; Millan, M.J.; Marin, P. 5-HT(6) receptor recruitment of mTOR as a mechanism for perturbed cognition in schizophrenia. *EMBO Mol Med*. **2012**, 4(10), 1043-1056.
12. Bockaert, J.; Bécamel, C.; Chaumont-Dubel, S.; Claeysen, S.; Vandermoere, F.; Marin, P. Novel and atypical pathways for serotonin signaling. *Fac Rev.* **2021**, 10, 52.
13. Martin, P.Y.; Doly, S.; Hamieh, A.M.; Chapuy, E.; Canale V.; Drop, M.; Chaumont-Dubel, S.; Bantreil, X.; Lamaty, F.; Bojarski, A.J.; Zajdel, P.; Eschalier, A.; Marin, P.; Courteix, C. mTOR activation by constitutively active serotonin6 receptors as new paradigm in neuropathic pain and its treatment. Prog Neurobiol. 2020 Oct;193:101846.
14. Chaumont-Dubel S, Dupuy V, Bockaert J, Bécamel C, Marin P. The 5-HT6 receptor interactome: New insight in receptor signaling and its impact on brain physiology and pathologies. Neuropharmacology 2020, 172, 107839.
15. Vanda, D.; Canale, V.; Chaumont-Dubel, S.; Kurczab, R.; Satała G.; Koczurkiewicz-Adamczyk, P.; Krawczyk, M.; Pietruś, W.; Blicharz, K.; Pękala, E.; Bojarski, AJ.; Popik, P.; Marin, P.; Soural, M.; Zajdel P. Imidazopyridine-Based 5-HT6 Receptor Neutral Antagonists: Impact of N1-Benzyl and N1-Phenylsulfonyl Fragments on Different Receptor Conformational States. J Med Chem. 2021 Jan 28;64(2):1180-1196.
16. Drop, M.; Jacquot, F.; Canale, V.; Chaumont-Dubel, S.; Walczak, M.; Satała, G.; Nosalska, K.; Mahoro, GU.; Słoczyńska, K.; Piska, K.; Lamoine, S.; Pękala, E.; Masurier, N.; Bojarski, AJ.; Pawłowski, M.; Martinez, J.; Subra, G.; Bantreil, X.; Lamaty, F.; Eschalier, A.; Marin, P.; Courteix, C.; Zajdel, P. Neuropathic pain-alleviating activity of novel 5-HT6 receptor inverse agonists derived from 2-aryl-1H-pyrrole-3-carboxamide. *Bioorg Chem.* **2021**, 115, 105218.
17. Yun, H.M.; Kim, S.; Kim, H.J.; Kostenis, E.; Kim, J.I.; Seong, J.Y.; Baik, J.H.; Rhim, H. The novel cellular mechanism of human 5-HT6 receptor through an interaction with Fyn. *J Biol Chem.* **2007**, 282(8), 5496-5505.
18. Kim, S.H.; Kim, D.H.; Lee, K.H.; Im, S.K.; Hur, E.M.; Chung, K.C.; Rhim, H. Direct interaction and functional coupling between human 5-HT6 receptor and the light chain 1 subunit of the microtubule-associated protein 1B (MAP1B-LC1). *PLoS One.* **2014**, 9(3), e91402.
19. Yun, H.M.; Baik, J.H.; Kang, I.; Jin, C.; Rhim, H. Physical interaction of Jab1 with human serotonin 6 G-protein-coupled receptor and their possible roles in cell survival. *J Biol Chem.* **2010**, 285(13), 10016-10029.
20. Canale, V.; Trybała, W.; Chaumont-Dubel, S.; KoczurkiewiczAdamczyk, P.; Satała, G.; Bento, O.; Blicharz-Futera, K.; Bantreil, X.; Pękala, E.; Bojarski, A.J.; Lamaty, F.;, Marin, P.; Zajdel, P. 1-(Arylsulfonyl-isoindol-2-yl)piperazines as 5-HT6R Antagonists: Mechanochemical Synthesis, In Vitro Pharmacological Properties and Glioprotective Activity. *Biomolecules* **2023**, 13, 12.

21. Kucwaj-Brysz, K.; Baltrukevich, H.; Czarnota, K.; Handzlik, J. Chemical update on the potential for serotonin 5-HT6 and 5-HT7 receptor agents in the treatment of Alzheimer's disease. *Bioorg Med Chem Lett.* **2021**, 49, 128275.
22. Liu, K.G.; Robichaud, A.J. 5-HT6 medicinal chemistry. *Int Rev Neurobiol*. **2010**, 94, 1-34.
23. Grychowska, K.; Satała, G.; Kos, T.; Partyka, A.; Colacino, E.; Chaumont-Dubel, S.; Bantreil, X.; Wesołowska, A.; Pawłowski, M.; Martinez, J.; Marin, P.; Subra, G.; Bojarski, AJ.; Lamaty, F.; Popik, P.; Zajdel, P. Novel 1H-Pyrrolo[3,2-c]quinoline Based 5-HT6 Receptor Antagonists with Potential Application for the Treatment of Cognitive Disorders Associated with Alzheimer's Disease. *ACS Chem Neurosci.* **2016**, 7(7), 972-83.
24. Wesołowska, A. Potential role of the 5-HT6 receptor in depression and anxiety: an overview of preclinical data. *Pharmacol Rep*. **2010**, 62(4), 564-77.
25. Woods, S.; Clarke, N.N.; Layfield, R.; Fone, K.C. 5-HT(6) receptor agonists and antagonists enhance learning and memory in a conditioned emotion response paradigm by modulation of cholinergic and glutamatergic mechanisms. *Br J Pharmacol*. **2012**, 167(2), 436-449.
26. Ivachtchenko, A.V.; Lavrovsky, Y.; Ivanenkov, Y.A. AVN-211, Novel and Highly Selective 5-HT6 Receptor Small Molecule Antagonist, for the Treatment of Alzheimer's Disease. *Mol Pharm.* **2016**, 13(3), 945-963.
27. Comery, TA.; Aschmies, S.; Haydar, S.; Hughes, Z.; Huselton, C.; Kowal, D.; Kramer, A.; McFarlane, G.; Monaghan, M.; Smith, D.; Zhang, G.; Zhang, J.; Zhang, M.; Reinhart, P.; Robichaud AJ.;. Pangalos MN. SAM-531, N,N-dimethyl-3-{[3-(1-naphthylsulfonyl)-1H-indazol-5-yl]oxy} propan-1-amine, a novel serotonin-6 receptor antagonist with preclinical pro-cognitive efficacy. *Alzheimers Dement*. **2010**, S548-S549.
28. Arnt, J.; Bang-Andersen, B.; Grayson, B.; Bymaster, F.P.; Cohen, M.P.; DeLapp, N.W.; Giethlen, B.; Kreilgaard, M.; McKinzie, D.L.; Neill, J.C.; Nelson, D.L.; Nielsen, S.M.; Poulsen, M.N.; Schaus, J.M.; Witten, L.M. Lu AE58054, a 5-HT6 antagonist, reverses cognitive impairment induced by subchronic phencyclidine in a novel object recognition test in rats. *Int J Neuropsychopharmacol*. **2010**, 13(8), 1021-1033.
29. Upton, N.; Chuang, T.T.; Hunter, A.J.; Virley, D.J. 5-HT6 receptor antagonists as novel cognitive enhancing agents for Alzheimer's disease. *Neurotherapeutics* **2008**, 5(3), 458-469.
30. Schaffhauser, H.; Mathiasen, J.R.; Dicamillo, A.; Huffman, M.J.; Lu, L.D.; McKenna, B.A.; Qian, J.; Marino, M.J. Dimebolin is a 5-HT6 antagonist with acute cognition enhancing activities. *Biochem Pharmacol*. **2009**, 78(8), 1035-1042.
31. Bachurin, S.; Bukatina, E.; Lermontova, N.; Tkachenko, S.; Afanasiev, A.; Grigoriev, V.; Grigorieva, I.; Ivanov, Y.; Sablin, S.; Zefirov, N. Antihistamine agent Dimebon as a novel neuroprotector and a cognition enhancer. *Ann N Y Acad Sci*. **2001**, 939, 425-35.
32. Wu, J.; Li, Q.; Bezprozvanny, I. Evaluation of Dimebon in cellular model of Huntington's disease. *Mol Neurodegener*. **2008**, 3, 15.
33. Grigorev, V.V.; Dranyi, O.A.; Bachurin, S.O. Comparative study of action mechanisms of dimebon and memantine on AMPA- and NMDA-subtypes glutamate receptors in rat cerebral neurons. *Bull Exp Biol Med*. **2003**, 136(5), 474-477.
34. Lermontova, N.N.; Redkozubov, A.E.; Shevtsova, E.F.; Serkova, T.P.; Kireeva, E.G.; Bachurin, S.O. Dimebon and tacrine inhibit neurotoxic action of beta-amyloid in culture and block L-type Ca(2+) channels. *Bull Exp Biol Med*. **2001**, 132(5), 1079-1083.
35. Bachurin, S.O.; Shevtsova, E.P.; Kireeva, E.G.; Oxenkrug, G.F.; Sablin, S.O. Mitochondria as a target for neurotoxins and neuroprotective agents. *Ann N Y Acad Sci*. **2003**, 993, 334-344.
36. Nirogi, R.; Shinde, A.; Kambhampati, R.S.; Mohammed, A.R.; Saraf, S.K.; Badange, R.K.; Bandyala, T.R.; Bhatta, V.; Bojja, K.; Reballi, V.; Subramanian, R.; Benade, V.; Palacharla, R.C.; Bhyrapuneni, G.; Jayarajan, P.; Goyal, V.; Jasti, V. Discovery and Development of 1-[(2-Bromophenyl)sulfonyl]-5-methoxy-3-[(4-methyl-1-piperazinyl)methyl]-1H-indole Dimesylate Monohydrate (SUVN-502): A Novel.; Potent.; Selective and Orally Active Serotonin 6 (5-HT6) Receptor Antagonist for Potential Treatment of Alzheimer's Disease. *J Med Chem*. **2017**, 60(5), 1843-1859.
37. Bell, J.; Baird-Bellaire, S.; Leil, T.; Comery, T.; Plotka, A.; Antinew, J.; Vandal, G.; Chalon, S.; James, K. Single- and multiple-dose safety, tolerability and pharmacokinetics of a Novel 5HT6 receptor full antagonist (SAM-760) for the treatment of the symptoms of Alzheimer's disease in healthy young adults and elderly subjects. *Alzheimers Dement*. **2012**, S778-S778.
38. Comery, T.; Zasadny, K.; Morris, E.; Antinew, J.; Bell, J.; Billing, B.; Boyden, T.; Esterlis, I.; Huang, Y.; Kupiec, J.; Kuszpit, K.; Leil, T.; Nabulsi, N.; Planeta-Wilson, B.; Plotka, A.; Skaddan, M.; Vandal, G.; Carson, R.; Katz, E.; McCarthy, T. Receptor occupancy of the 5-HT6 receptor antagonist SAM-760 in non-human primates and healthy human volunteers. *Alzheimers Dement*. **2012**, S794-S795.

39. Schmidt, E.; Areberg, J.; Evans, P.; Zann, V.; Søgaard, B. Assessment of the Contribution of CYP2D6 to the Elimination of Idalopirdine as well as the Absolute Bioavailability Following Multiple Oral Dosing. *ASCPT 2016 Annual Meeting, San Diego, CA, US, March 8-12*, **2016**.
40. Galimberti, D.; Scarpini, E. Idalopirdine as a treatment for Alzheimer's disease. *Expert Opin Investig Drugs.* **2015**, 24(7), 981-987.
41. Arnt, J.; Olsen, C.K. 5-HT6 receptor ligands and their antipsychotic potential. *Int Rev Neurobiol.* **2011**, 96, 141-161.
42. Mørk, A.; Russell, R.V.; de Jong, IE.; Smagin, G. Effects of the 5-HT6 receptor antagonist idalopirdine on extracellular levels of monoamines.; glutamate and acetylcholine in the rat medial prefrontal cortex. *Eur J Pharmacol*. **2017**, 799, 1-6.
43. Ferris, C.F.; Kulkarni, P.; Yee, J.R.; Nedelman, M.; de Jong, I.E.M. The Serotonin Receptor 6 Antagonist Idalopirdine and Acetylcholinesterase Inhibitor Donepezil Have Synergistic Effects on Brain Activity-A Functional MRI Study in the Awake Rat. *Front Pharmacol*. **2017**, 8, 279.
44. Amat-Foraster, M.; Leiser, S.C.; Herrik, K.F.; Richard, N.; Agerskov, C.; Bundgaard, C.; Bastlund, J.F.; de Jong, I.E.M. The 5-HT6 receptor antagonist idalopirdine potentiates the effects of donepezil on gamma oscillations in the frontal cortex of anesthetized and awake rats without affecting sleep-wake architecture. *Neuropharmacology* **2017**,113, 45-59.
45. Chuang, A.T.T.; Foley, A.; Pugh, P.L.; Sunter, D.; Tong, X.; Regan, C.; Dawson, L.A.; Medhurst, A.D.; Upton, N. 5-HT6 receptor antagonist SB-742457 as a novel cognitive enhancing agent for Alzheimer's disease. *Alzheimers Dement*. **2006**, 2, S631–S632.
46. Dawson, L.A. The central role of 5-HT6 receptors in modulating brain neurochemistry. *Int Rev Neurobiol.* **2011**, 96, 1-26.
47. Sabbagh, M.N.; Shill, H.A. Latrepirdine, a potential novel treatment for Alzheimer's disease and Huntington's chorea. Curr Opin Investig Drugs. 2010 Jan;11(1):80-91.
48. Chew, M.L.; Mordenti, J.; Yeoh, T.; Ranade, G.; Qiu, R.; Fang, J.; Liang, Y.; Corrigan, B. Minimization of CYP2D6 Polymorphic Differences and Improved Bioavailability via Transdermal Administration: Latrepirdine Example. *Pharm Res.* **2016**, 33(8), 1873-1880.
49. Santos, J.; Lobato, L.; Vale, N. Clinical pharmacokinetic study of latrepirdine via in silico sublingual administration. *In Silico Pharmacol.* **2021**, 9(1), 29.
50. Giorgetti, M.; Gibbons, J.A.; Bernales, S.; Alfaro, I.E.; Drieu, La Rochelle, C.; Cremers, T.; Altar, C.A.; Wronski, R.; Hutter-Paier, B.; Protter, A.A. Cognition-enhancing properties of Dimebon in a rat novel object recognition task are unlikely to be associated with acetylcholinesterase inhibition or N-methyl-D-aspartate receptor antagonism. *J Pharmacol Exp Ther.* **2010**, 333(3), 748-757.
51. Nirogi, R.; Mudigonda, K.; Bhyrapuneni, G.; Muddana, N.R.; Goyal, V.K.; Pandey, S.K.; Palacharla, R.C. Safety, Tolerability and Pharmacokinetics of the Serotonin 5-HT6 Receptor Antagonist, SUVN-502, in Healthy Young Adults and Elderly Subjects. *Clin Drug Investig.* **2018**, 38(5), 401-415.
52. Nirogi, R.; Abraham, R.; Benade, V.; Medapati, R.B.; Jayarajan, P.; Bhyrapuneni, G.; Muddana, N.; Mekala, V.R.; Subramanian, R.; Shinde, A.; Kambhampati, R.; Jasti V. SUVN-502, a novel, potent, pure, and orally active 5-HT6 receptor antagonist: pharmacological, behavioral, and neurochemical characterization. *Behav Pharmacol.* **2019**, 30(1), 16-35.
53. Nirogi, R.; Jayarajan, P.; Abraham, R.; Tadiparthi, J.; Benade, V.; Shinde, A.K.; Mohammed, A.R.; Badange, R.K.; Goyal, V.K.; Palacharla, V.R.C.; Ravula, J.; Jetta, S.; Subramanian, R. Effects of Masupirdine (SUVN-502) on Agitation/Aggression and Psychosis in Patients with Probable Alzheimer's Disease: A Post Hoc Analysis. *Alzheimer's Association International Conference* 2022, P3-001.
54. Sawant-Basak, A.; Obach, RS.; Doran, A.; Lockwood, P.; Schildknegt, K.; Gao, H.; Mancuso, J.; Tse, S.; Comery, T.A. Metabolism of a 5HT6 Antagonist, 2-Methyl-1-(Phenylsulfonyl)-4-(Piperazin-1-yl)-1H-Benzo[d]imidazole (SAM-760): Impact of Sulfonamide Metabolism on Diminution of a Ketoconazole-Mediated Clinical Drug-Drug Interaction. *Drug Metab Dispos.* **2018**, 46(7), 934-942
55. Morozova, M.A.; Lepilkina, T.A.; Rupchev, G.E.; Beniashvily, A.G.; Burminskiy, D.S.; Potanin, SS.; Bondarenko, E.V.; Kazey, V.I.; Lavrovsky, Y.; Ivachtchenko, A.V. Add-on clinical effects of selective antagonist of 5HT6 receptors AVN-211 (CD-008-0173) in patients with schizophrenia stabilized on antipsychotic treatment: pilot study. *CNS Spectr*. **2014**, 19(4), 316-323.
56. https://www.biospace.com/article/releases/avineuro-completed-phase-ii-clinical-study-of-avn-211-a-selective-5-ht6-receptor-antagonist-/

57. Morozova, M.; Burminskiy, D.; Rupchev, G.; Lepilkina, T.; Potanin, S.; Beniashvili, A.; Lavrovsky, Y.; Vostokova, N.; Ivaschenko, A. 5-HT6 Receptor Antagonist as an Adjunct Treatment Targeting Residual Symptoms in Patients With Schizophrenia: Unexpected Sex-Related Effects (Double-Blind Placebo-Controlled Trial). *J Clin Psychopharmacol.* **2017**, 37(2), 169-175.
58. Brisard, C.; Safirstein, B.; Booth, K.; Hua, L.; Brault, Y.; Raje, S.; Leventer, S. Safety, tolerability, and preliminary efficacy of SAM-531, a 5HT-6 antagonist, in subjects with mild-to-moderate Alzheimer's disease: Results from a phase 2a study. *Alzheimers Dement*. **2010**, S311-S311.
59. NCT00895895 - Study Comparing 3 Dosage Levels Of SAM-531 In Outpatients With Mild To Moderate Alzheimer Disease
60. WO2014037532 - Methods of treating Alzheimer’s disease and pharmaceutical compositions thereof
61. https://www.biospace.com/article/releases/saegis-pharmaceuticals-inc-completes-phase-iia-clinical-study-of-sgs518-/
62. Wilkinson, D.; Windfeld, K.; Colding-Jørgensen, E. Safety and efficacy of idalopirdine, a 5-HT6 receptor antagonist, in patients with moderate Alzheimer's disease (LADDER): a randomised, double-blind, placebo-controlled phase 2 trial. *Lancet Neurol*. **2014**, 13(11), 1092-1099.
63. Atri, A.; Frölich, L.; Ballard, C.; Tariot, P.N.; Molinuevo, J.L.; Boneva, N.; Windfeld, K.; Raket, L.L.; Cummings, J.L. Effect of Idalopirdine as Adjunct to Cholinesterase Inhibitors on Change in Cognition in Patients With Alzheimer Disease: Three Randomized Clinical Trials. *JAMA*. **2018**, 319(2), 130-142.
64. Maher-Edwards, G.; Zvartau-Hind, M.; Hunter, A.J.; Gold, M.; Hopton, G.; Jacobs, G.; Davy, M.; Williams, P. Double-blind, controlled phase II study of a 5-HT6 receptor antagonist, SB-742457, in Alzheimer's disease. *Curr Alzheimer Res*. **2010**, 7(5), 374-385.
65. Maher-Edwards, G.; Dixon, R.; Hunter, J.; Gold, M.; Hopton, G.; Jacobs, G.; Hunter, J.; Williams, P. SB-742457 and donepezil in Alzheimer disease: a randomized.; placebo-controlled study. *Int J Geriatr Psychiatry*. **2011**, 26(5), 536-544.
66. Maher-Edwards, G.; Watson, C.; Ascher, J.; Barnett, C.; Boswell, D.; Davies, J.; Fernandez, M.; Kurz, A.; Zanetti, O.; Safirstein, B.; Schronen, JP.; Zvartau-Hind, M.; Gold, M. Two randomized controlled trials of SB742457 in mild-to-moderate Alzheimer's disease. *Alzheimers Dement (N Y)*. **2015**, 1(1), 23-36.
67. Lang, F.M.; Mo, Y.; Sabbagh, M.; Solomon, P.; Boada, M.; Jones, R.W.; Frisoni, G.B.; Grimmer, T.; Dubois, B.; Harnett, M.; Friedhoff, S.R.; Coslett, S.; Cummings, J.L. Intepirdine as adjunctive therapy to donepezil for mild-to-moderate Alzheimer's disease: A randomized.; placebo-controlled.; phase 3 clinical trial (MINDSET). *Alzheimers Dement (N Y)*. **2021**, 7(1), e12136.
68. Lang, FM.; Kwon, DY.; Aarsland, D.; Boeve, B.; Tousi, B.; Harnett, M.; Mo, Y.; Noel Sabbagh, M. An international.; randomized.; placebo-controlled.; phase 2b clinical trial of intepirdine for dementia with Lewy bodies (HEADWAY-DLB). *Alzheimers Dement (N Y)*. **2021**, 7(1), e12171.
69. Fernandez, H.H. SYN120 (a dual 5-HT6/5-HT2A antagonist) study to evaluate safety, tolerability, and efficacy in Parkinson’s disease dementia (SYNAPSE): Phase 2a study results. *Neurology* **2019**, 92 (15), S4.005.
70. Morozova, M.A.; Beniashvili, A.G.; Lepilkina, T.A.; Rupchev, G.E. Double-blind placebo-controlled randomized efficacy and safety trial of add-on treatment of dimebon plus risperidone in schizophrenic patients during transition from acute psychotic episode to remission. *Psychiatr Danub.* **2012**, 24(2), 159-66.
71. Doody, R.S.; Gavrilova, S.I.; Sano, M.; Thomas, R.G.; Aisen, P.S.; Bachurin, S.O.; Seely, L.; Hung, D; dimebon investigators. Effect of dimebon on cognition, activities of daily living, behaviour, and global function in patients with mild-to-moderate Alzheimer's disease: a randomised, double-blind, placebo-controlled study. *Lancet* **2008**, 372(9634), 207-215.
72. https://www.pfizer.com/news/press-release/press-release-detail/pfizer_and_medivation_announce_results_from_two_phase_3_studies_in_dimebon_latrepirdine_alzheimer_s_disease_clinical_development_program)
73. Nirogi, R.; Ieni, J.; Goyal, V.K.; Ravula, J.; Jetta, S.; Shinde, A.; Jayarajan, P.; Benade, V.; Palacharla, VRC.; Dogiparti, DK.; Jasti, V.; Atri, A.; Cummings, J. Effect of masupirdine (SUVN-502) on cognition in patients with moderate Alzheimer's disease: A randomized, double-blind, phase 2, proof-of-concept study. *Alzheimers Dement (N Y)*. **2022**, 8(1), e12307.
74. Nirogi, R.; Goyal, V.K.; Benade, V.; Subramanian, R.; Ravula, J.; Jetta, S.; Shinde, A.; Pandey, S.K.; Jayarajan, P.; Jasti V.; Cummings, J. Effect of Concurrent Use of Memantine on the Efficacy of Masupirdine (SUVN-502): A Post Hoc Analysis of a Phase 2 Randomized Placebo-Controlled Study. *Neurol Ther*. 2022, 11(4), 1583-1594.

75. Nirogi, R.; Jayarajan, P.; Benade, V.; Shinde, A.; Goyal, V.K.; Jetta, S.; Ravula, J.; Abraham, R.; Grandhi, V.R.; Subramanian, R.; Pandey, S.K.; Badange, R.K.; Mohammed, A.R.; Jasti, V.; Ballard, C.; Cummings, J. Potential beneficial effects of masupirdine (SUVN-502) on agitation/aggression and psychosis in patients with moderate Alzheimer's disease: Exploratory post hoc analyses. *Int J Geriatr Psychiatry*. **2022**, 37(10).
76. NCT05397639 - Masupirdine for the Treatment of Agitation in Dementia of the Alzheimer's Type
77. Fullerton, T.; Binneman, B.; David, W.; Delnomdedieu, M.; Kupiec, J.; Lockwood, P.; Mancuso, J.; Miceli, J.; Bell, J. A Phase 2 clinical trial of PF-05212377 (SAM-760) in subjects with mild to moderate Alzheimer's disease with existing neuropsychiatric symptoms on a stable daily dose of donepezil. *Alzheimers Res Ther*. **2018**, 10(1), 38.
78. Bourson, A.; Borroni, E.; Austin, R.H.; Monsma, F.J Jr.; Sleight, A.J. Determination of the role of the 5-ht6 receptor in the rat brain: a study using antisense oligonucleotides. *J Pharmacol Exp Ther*. **1995**, 274(1), 173-180.
79. Mitchell, E.S. 5-HT6 receptor ligands as antidementia drugs. *Int Rev Neurobiol.* **2011**, 96, 163-187.
80. Rossé, G.; Schaffhauser, H. 5-HT6 receptor antagonists as potential therapeutics for cognitive impairment. *Curr Top Med Chem*. **2010**, 10(2), 207-221.
81. Johnson, C.N.; Ahmed, M.; Miller, N.D. 5-HT6 receptor antagonists: prospects for the treatment of cognitive disorders including dementia. *Curr Opin Drug Discov Devel*. **2008**, 11(5), 642-654.
82. Rogers, J.; Polhamus, D.; Lockwood, P.; Brault, Y.; Desmet, A.; Ito, K.; Romero, K.; Qiu, R.; Corrigan, B.; Gillespie, W.; Gastonguay, M. Model-based analysis to support strategic decision-making: A case study from the development of a 5HT6 antagonist for the treatment of Alzheimer's disease. *Alzheimers Dement*. **2012**, P585-P585.
83. Atri, A.; Tong, G.; Isojarvi, J.; Odergren, T. A 5-HT6 antagonist as adjunctive therapy to cholinesterase inhibitors in patients with mild to-moderate Alzheimer's disease: idalopirdine in phase III. *J Am Geriatr Soc. Conference: American Geriatrics Society* **2016** Annual Scientific Meeting. Long beach.; CA United States.
84. Parker, C.A.; Rabiner, E.A.; Gunn, R.N.; Searle, G.; Martarello, L.; Comley, R.A.; Davy, M.; Wilson, A.A.; Houle, S.; Mizrahi, R.; Laruelle, M.; Cunningham, V.J. Human Kinetic Modeling of the 5HT6 PET Radioligand 11C-GSK215083 and Its Utility for Determining Occupancy at Both 5HT6 and 5HT2A Receptors by SB742457 as a Potential Therapeutic Mechanism of Action in Alzheimer Disease. *J Nucl Med*. **2015**, 56(12), 1901-1909.
85. Weintraub, D. Impact of SYN120 (a Dual 5-HT6/5-HT2A antagonist) on psychiatric symptoms in patients with Parkinson's disease dementia: results from a Phase 2a Study. *Mov Disord*. **2018**, 33, S105.
86. Lockwood, P.; Bell, J.; Chen, I.; Miceli, J.; Macci, K.; Van Winkle, J.; Planeta, B.; Henry, S.; Nabulsi, N.; Carson, R. 5HT2a receptor occupancy (RO) in healthy subjects determined by positron emission tomography (PET) following single-dose administration of SAM760 (PF-05212377). *Clin Pharmacol Ther*. **2015**, 97, S21.
87. Lacroix, L.P.; Dawson, L.A.; Hagan, J.J.; Heidbreder, C.A. 5-HT6 receptor antagonist SB-271046 enhances extracellular levels of monoamines in the rat medial prefrontal cortex. *Synapse* **2004**, 51(2), 158-164.
